# Articular involvement in childhood Familial Mediterranean Fever

**DOI:** 10.1186/1546-0096-13-S1-P96

**Published:** 2015-09-28

**Authors:** K Barut, AB Sinoplu, G Yucel, G Pamuk, A Adrovic, S Sahin, O Kasapcopur

**Affiliations:** 1 Istanbul University, Cerrahpasa Medical Faculty, Pediatric Rheumatology, Istanbul, Turkey

## Introduction

Familial Mediterranean fever is an autosomal recessively inherited autoinflammatory disease which is clinically manifested with periodic episodes of fever, serositis and arthritis. Articular involvement is also a frequent presentation after fever and peritonitis in childhood FMF.

## Objective

The aim of this study is to evaluate the demographic and clinical features of the common articular findings in the course of FMF and compare these with the relatively rare occasions of chronic arthritis and FMF comorbidity.

## Patients and results

Among the 708 patients diagnosed with FMF; arthritis was defined in 288 (40,7%) (female/male 150/138) cases. In 192 (66%) children the affected joints were observed primarily as the ankles, subsequently followed by knees in 148 (51%) cases. The incidence of the rare articular involvements were respectively, the elbows in 14 (4,8%) children, the wrists in 11 (3,8%) children and the hip joint with the rate of %2,7 of all cases. The shoulder girdle was affected only in one patient. Exertional leg pain was reported by 467 (66%) patients. Erysipelas like erythema coincidentally found in 213 (73%) cases and in 26 (3,7%) patients enthesitis was detected. Articular findings were mainly acute monoarthritis without leading to sequelae.

When the children presented with arthritis were considered, the mean age at disease onset was estimated to be 5,2 ± 3,7 years and the mean age at diagnosis was 8,1±4 years.

The analysis of MEFV genetic mutation of the patients with arthritis also resulted mainly as homozygote M694V mutations with the rate of 104(36,1%); heterozygote M694V mutations in 52 (18%) and M694V/M680I compound heterozygote in 26(9%) counting for the total of M694V mutations in 192 (66,6%) of cases. The rate of the rest of the mutations in exon 10 region was corresponded to 20(6,9%) and E148Q mutations, which is a common mutation in exon 2 region were found in 11(3,8%) of the all cases.

The mean duration of the episodes of FMF mainly manifested by articular symptoms was yielded as 97 ± 53,6 hours (with the median of 72 hours, ranging between 24-168 hours) and when compared with the cases primarily manifested with abdominal pain and fever that was declared to be 59,4±34,3 hours in a former study, a statistically significant difference was revealed (p<0,0001).

In 23 (8%) cases with the diagnosis of FMF and articular manifestations, a coincidental diagnosis of chronic arthritis was considered to be more consistent as the disease progressed. While 9 of the patients were turned out to have juvenile spondyloarthropathies (JSpA), 13 of them were identified as having oligoarticular juvenile idiopathic arthritis (JIA) and the remaining one was also diagnosed with seropositive polyarticular JIA. As 20 (%86) of those cases were presented with arthritis at the disease onset, with respect to the main clinical symptoms 6 (%26) cases of all showed episodes of abdominal pain and periodic fever at the same time. Among the ones with oligoarticular JIA, 7 (%30,4) of them were ANA positive and 5 of the 9 JSpA cases (%55,5) were found to be HLA B27 positive. MEFV genetic mutations of the children with the comorbidity of FMF and chronic arthritis were detected to be homozygote M694V in 5 (21,7%), M694V/M680I in 4(17,4%) and heterozygote M694V in 7(30,4%) of patients.

## Conclusion

Articular involvement in FMF is acute monoarthritis typically affecting the lower extremities in a periodic manner with short duration and without sequelae formation. The clinical entity of chronic arthritis and FMF coincidence can emerge as a rare occasion. Especially with the cases of oligoarticular JIA that show recurrence and quickly respond to treatment, the diagnosis of FMF should also be kept in mind.

